# Supplementary material for: Tracking Real-Time Changes in Working Memory Updating and Gating with the Event-Based Eye-Blink Rate
Source: Sci Rep. 2017 May 31;7:2547. doi: 10.1038/s41598-017-02942-3 (PMC5451427; doi:10.1038/s41598-017-02942-3)
Supplement: Supplementary file 1 — Supplementary Materials [file 41598_2017_2942_MOESM1_ESM.doc]

**Supplementary**

Tracking Real Time Changes in Working Memory Updating and Gating with the Event-Based Eye-Blink Rate

Rachel Rac-Lubashevsky, Heleen A. Slagter and Yoav Kessler

**Changes in the ebEBR throughout the course of the experiments**

The following analyses examined whether the ebEBR changed throughout the experiments, for example due to factors such as practice and fatigue. To this end, we sorted the trials within each condition, and binned them into 10 bins from the beginning of the experiment until the end (e.g., bin 1 = first 10% of the trials, bin 2 = second 10% of the trials etc.). A three-way ANOVA was conducted on the ebEBR data in each Experiment with Trial-Type (reference, comparison), Switching (switch, no-switch) and Bin (1-10) as within-subject independent variables.

Experiment 1

As reported in Experiment 1, the main effects of Trial- Type, *F(1,18)=9.07, MSe=.01, p=.007, η_p_^2^=.33* and Switching, *F(1,18)=24.69, MSe=.01, p<.001, η_p_^2^=.58,* were significant, but not the interaction between them, *F(1,18)=1.22, MSe=.01, p=.28, η_p_^2^=.06.* The main effect of Bin was not significant, *F(9,162)=.36, MSe=.04, p=.95, η_p_^2^=.02,* and neither were the two-way interactions with bin, not with Trial-Type, *F(9,162)=.1.20, MSe=.01, p=.29, η_p_^2^=.06,* nor with Switching, *F(9,162)=.1.21, MSe=.01, p=.29, η_p_^2^=.06*. The there-way interaction between Bin, Trial-Type and Switching was marginally significant, *F(9,162)=1.90, MSe=.01, p=.05, η_p_^2^=.09* (see Fig. S1). These results show that the effect of the ebEBR remained relatively constant throughout the experiment.


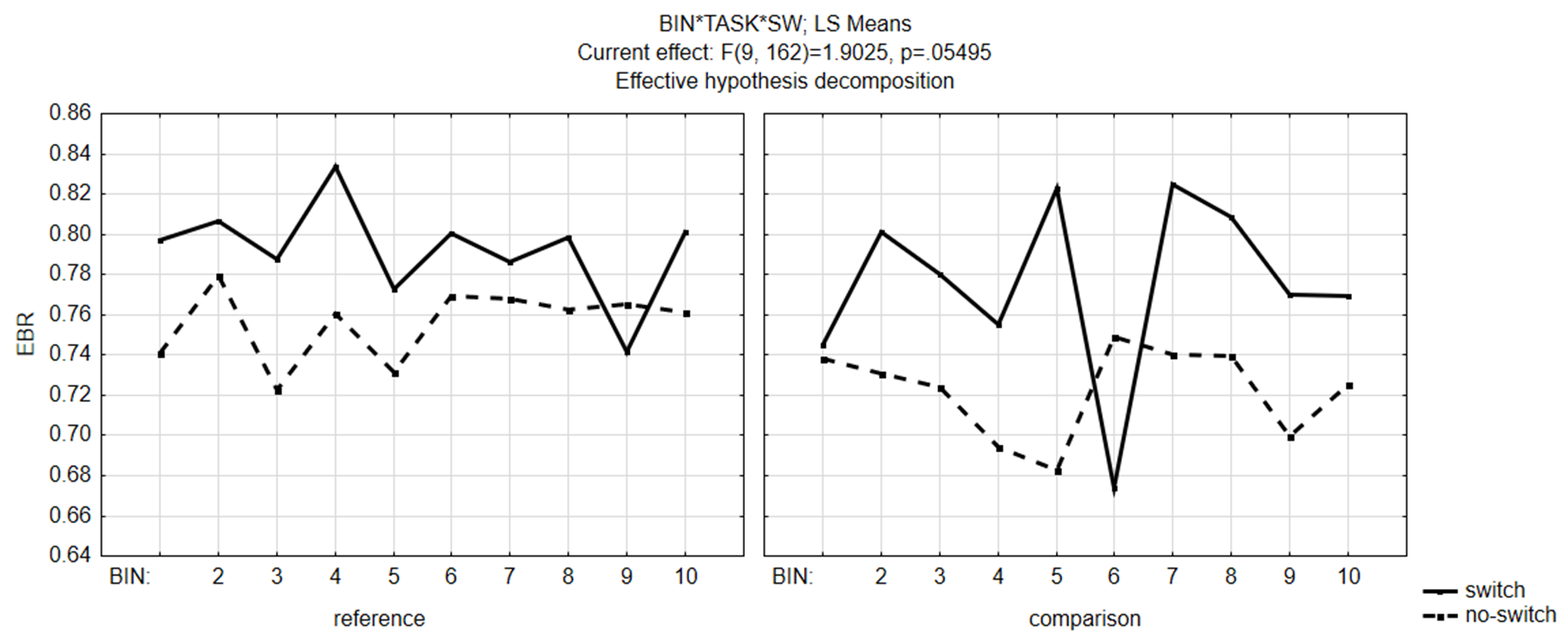


**Figure S1│ebEBR throughout the course of Experiment 1.** The ebEBR is presented as a function of time Trial-Type, Switching, and Bin. Note that the color mapping was switched between blocks 6 and 7, roughly corresponding to Bins 6-7.

Experiment 2

As reported in Experiment 2, the main effects of Trial-Type, *F(1,19)=5.70, MSe=.01, p=.03, η_p_^2^=.23* and Switching, *F(1,19)=14.03, MSe=.01, p=.001, η_p_^2^=.42*, were significant, but not the 2-way interaction between them, F*(1,19)*=.01, *MSe=.01, p=.93, η_p_^2^=.0004*. The main effect of Bin was also significant, *F(9,171)=5.05, MSe=.17, p<.001, η_p_^2^=.21* reflecting an increase in the ebEBR towards the end of the experiment (see Fig. S2). However, none of the interactions with Bin reached significance: Trial-Type and Bin, *F(9,171)=1.55, MSe=.01, p=.13, η_p_^2^=.07,* Switching and Bin, *F(9,171)=.51, MSe=.01, p=.87, η_p_^2^=.03,* nor the 3-way interaction, *F(9,171)=.57, MSe=.01, p=.82, η_p_^2^=.03.* Although the increase in the ebEBR could reflect overall practice or fatigue, it did not interact with the main effects of Trial-Type and Switching.


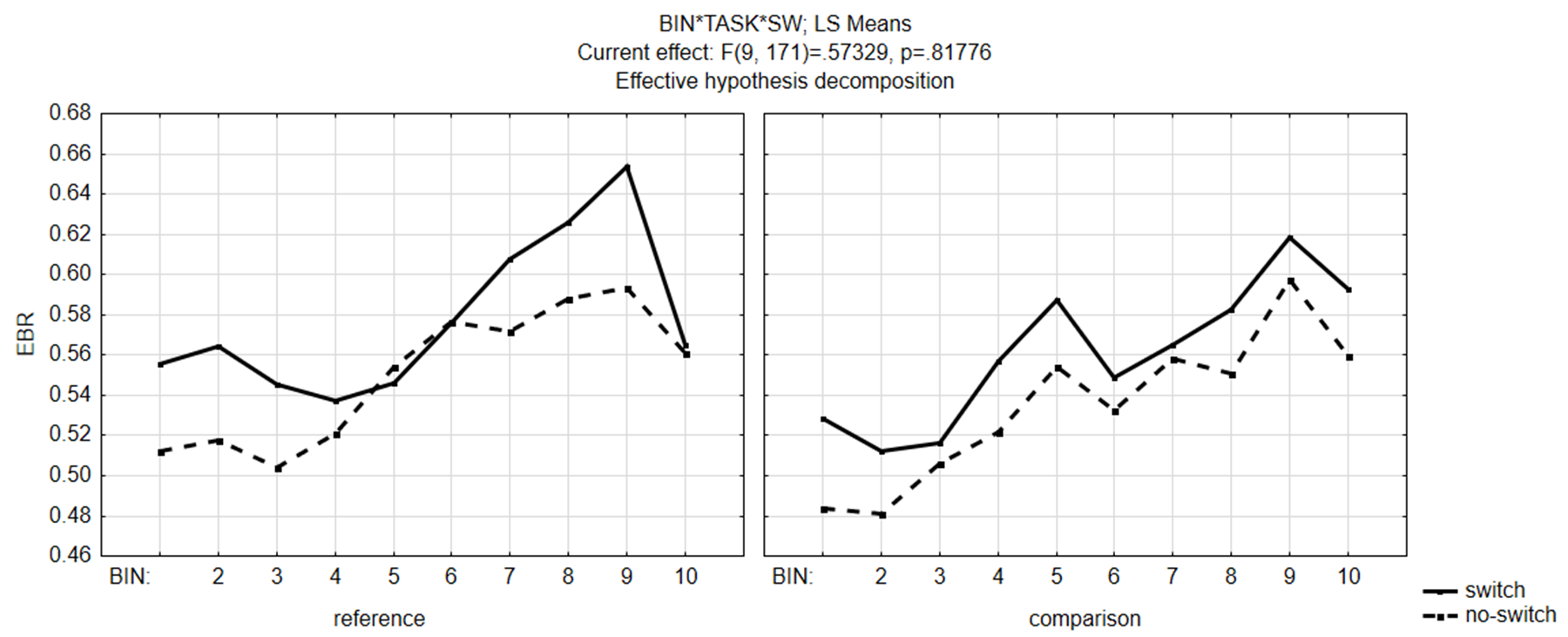


**Figure S2│ebEBR throughout the course of Experiment 2.** The ebEBR is presented as a function of Trial-Type, Switching, and Bin.

Experiment 3

Cue phase analysis

The main effect of Switching was significant, *F(1,20)=7.63, MSe=.007, p=.01, η_p_^2^=.28* and so was the main effect of Bin, *F(9,180)=6.72, MSe=.02, p<.001, η_p_^2^=.25.* Also, the two-way interaction between Switching and Trial-Type was significant, *F(1,20)=12.04, MSe=.009, p=.002, η_p_^2^=.37*. None of the interactions between Bin and the other variables reached significance: Trial-Type and Bin, *F(9,180)=.78, MSe=.01, p=.64, η_p_^2^=.0*4, and Switching and Bin, *F(9,180)=1.15, MSe=.01, p=.33, η_p_^2^=.0*5. The three-way interaction was also non-significant, *F(9,180)=1.35, MSe=.01, p=.21, η_p_^2^=.0*6 (see Fig. S3a).

Probe phase analysis

The main effects of Trial-Type *F(1,20)=9.29, MSe=.01, p=.01, η_p_^2^=.31* and Switching, *F(1,20)=7.56, MSe=.01, p=.01, η_p_^2^=.27* were significant, and so was their two-way interaction, *F(1,20)=4.36, MSe=.006, p=.05, η_p_^2^=.18*. The main effect of Bin was not significant, *F(9,180)=1.92, MSe=.03, p=.05, η_p_^2^=.0*9 and neither were the interactions between Bin and the other independent variables, not the two-way interaction between Trial-Type and Bin, *F(9,180)=1.41, MSe=.01, p=.19, η_p_^2^=.0*6 nor between Switching and Bin, *F(9,180)=1.70, MSe=.00, p=.09, η_p_^2^=.0*8. The three-way interaction was also not significant, *F(9,180)=1.68, MSe=.01, p=.10, η_p_^2^=.0*8 (see Fig. S3b).

Cue phase


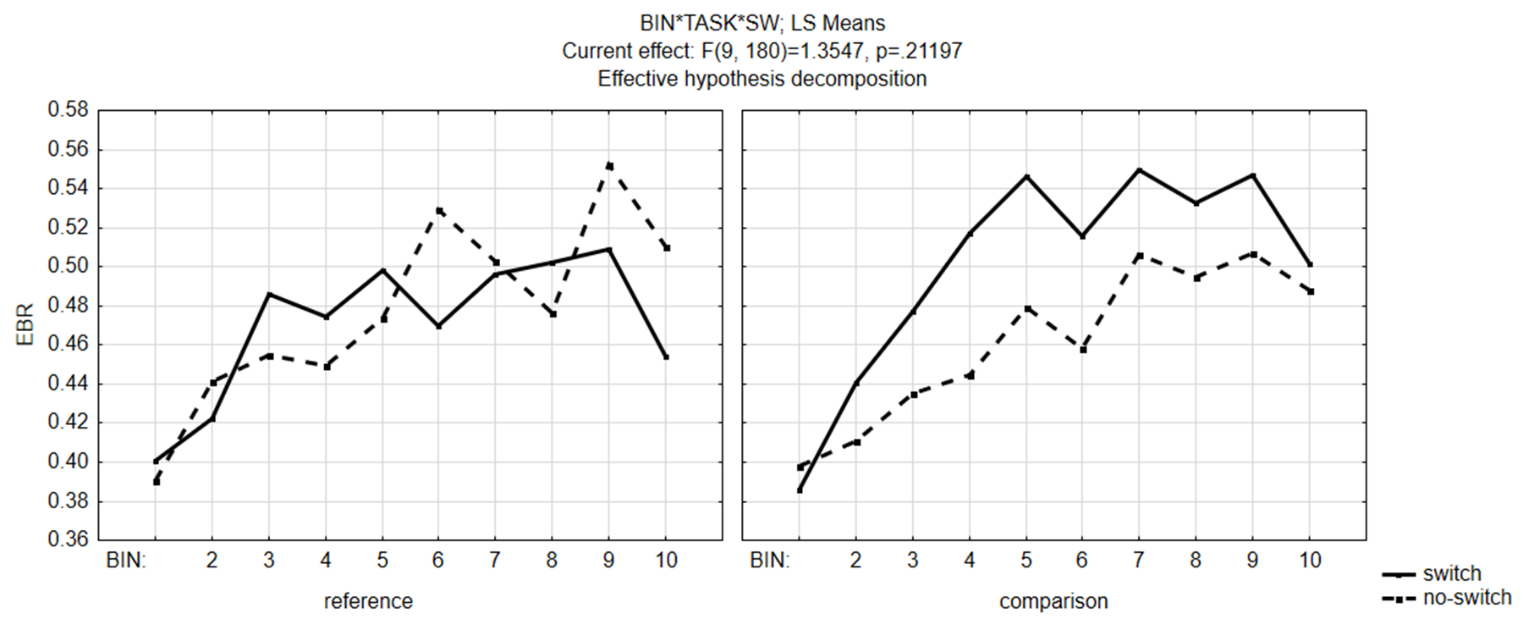


Probe phase


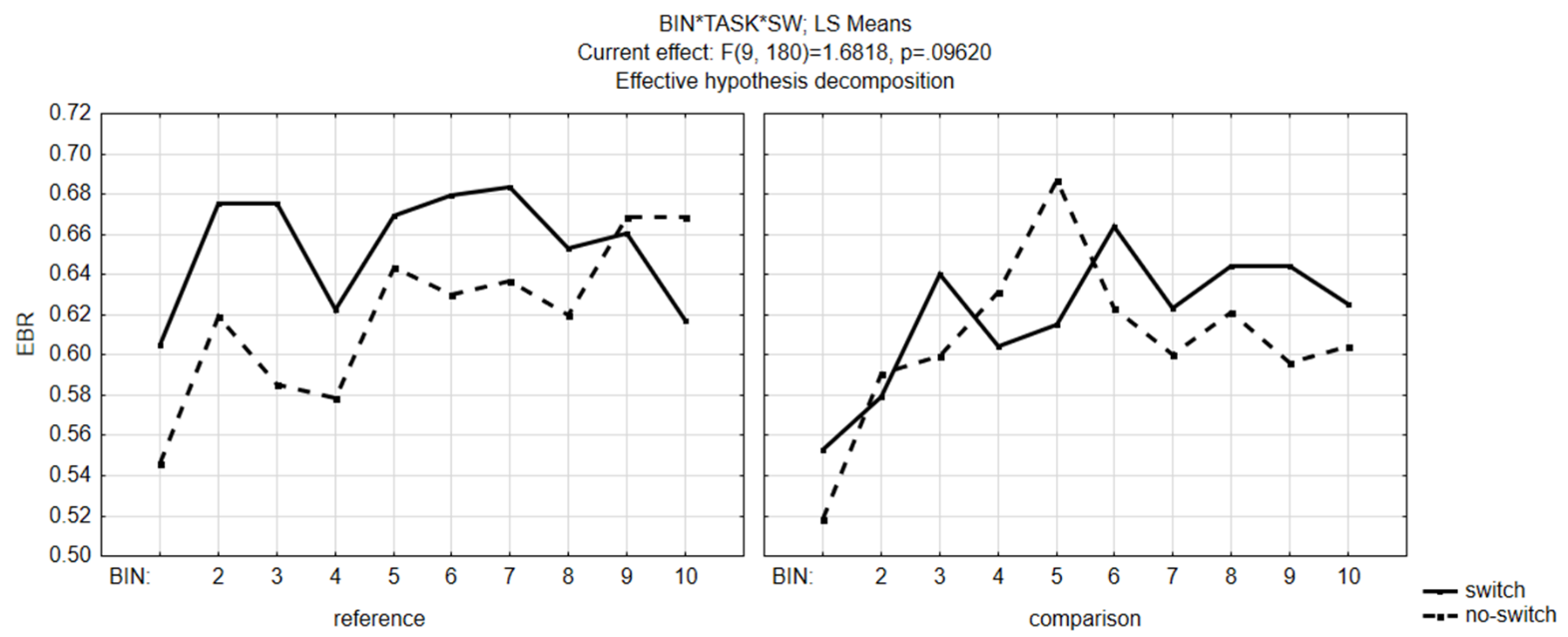


**Figure S3│ebEBR throughout the course of Experiment 3.** ebEBR is presented as a function of Trial-Type, Switching, and Bin. (a) Cue phase analysis (b) Probe phase analysis.

In conclusion, these results show that the effects of Trial-Type and Switching on the ebEBR are not limited to specific parts of the experiment and are not likely to be resulted from fatigue nor learning, which are only limited to the end or the beginning of the session, respectively.

**Cross-experiment analysis of the ebEBR**

A three-way ANOVA was conducted on the ebEBR data with Trial-Type (reference, comparison) and Switching (switch, no-switch) as within-subject independent variables and Experiment (1,2 and 3) as between-subject independent variable. In Experiment 3 only the stimulus locked window was analyzed. All the main effects were significant, *F(1,57)=32.00, MSe=.001, p<.001, η_p_^2^=.36* for Trial-Type, *F(1,57)=41.61, MSe=.001, p<.001, η_p_^2^=.42,* for Switching and, *F(2,57)=3.58, MSe=.23, p=.03, η_p_^2^=.11* for Experiment.

None of the interactions with Experiment were significant: Trial-Type and Experiment, *F(2,57)=.79, MSe=.001, p=.46, η_p_^2^=.03*, Switching and Experiment, *F(2,57)=1.95, MSe=.001, p=.15, η_p_^2^=.06,* and the 3-way interaction, *F(2,57)=1.49, MSe=.001, p=.23, η_p_^2^=.05.*

**Reliability of the ebEBR**

**Table S1│ Reliability.** Split-half reliability between odd and even trials of the ebEBR data was calculated and corrected using the Spearman-Brown formula.

**Descriptive statistics of ebEBR data**

**Table S2│** Descriptive statistics for the ebEBR data in all the conditions of the reference-back in Experiments 1-3.

**Behavior results**

Experiment 1

Pervious results using the reference-back paradigm^50,51^ demonstrate that (a) performance in reference trials is slower than in comparison trials, supporting the additional updating process required in the former, and (b) switching between the two trial types is associated with an additional cost, reflecting the time taken to open or close the gate to WM^52,53^. Our first aim was to replicate previous findings. A two-way ANOVA was conducted on the reaction time (RT) data with Trial-Type (reference, comparison) and Switching (switch, no-switch) as within-subject independent variables. Only correct trials preceded by 2 correct trials were included in this analysis. Trials in which RTs were more than 2 standard deviations from the mean of each condition for each participant were considered as outliers, as well as RTs faster than 200 ms (this trimming procedure excluded 3.98% of the trials). As predicted, slower RT was observed in reference trials than in comparison trials, *F(1,18)=42.26, MSe=4,055.44, p<.001, η_p_^2^=.70*. Also, RT were slower in switch trials compared to no-switch trials, *F(1,18)=56.19, MSe=7,158.55, p<.001, η_p_^2^=.76*. The two-way interaction was non-significant, *F(1,18)=.47, MSe=1,732, p=.50, η_p_^2^=.02* indicating that the switch cost was similar in both trial-types (see Fig. S4).

A parallel ANOVA was conducted on error proportions (PE). More errors were observed in reference than in comparison trials, *F(1,18)=27.39, MSe=.0018, p<.001, η_p_^2^=.60,* and also more errors were observed in switch than in no-switch trials, *F(1,18)=151.80, MSe=.0003, p<.001, η_p_^2^=.89,*. The two-way interaction was also significant, *F(1,18)=5.24, MSe=.0004, p=.03, η_p_^2^=.22*, reflecting a larger switch cost in reference trials than in comparison trials. Thus, at the behavioral level, the results in Experiment 1 replicated our previous findings.


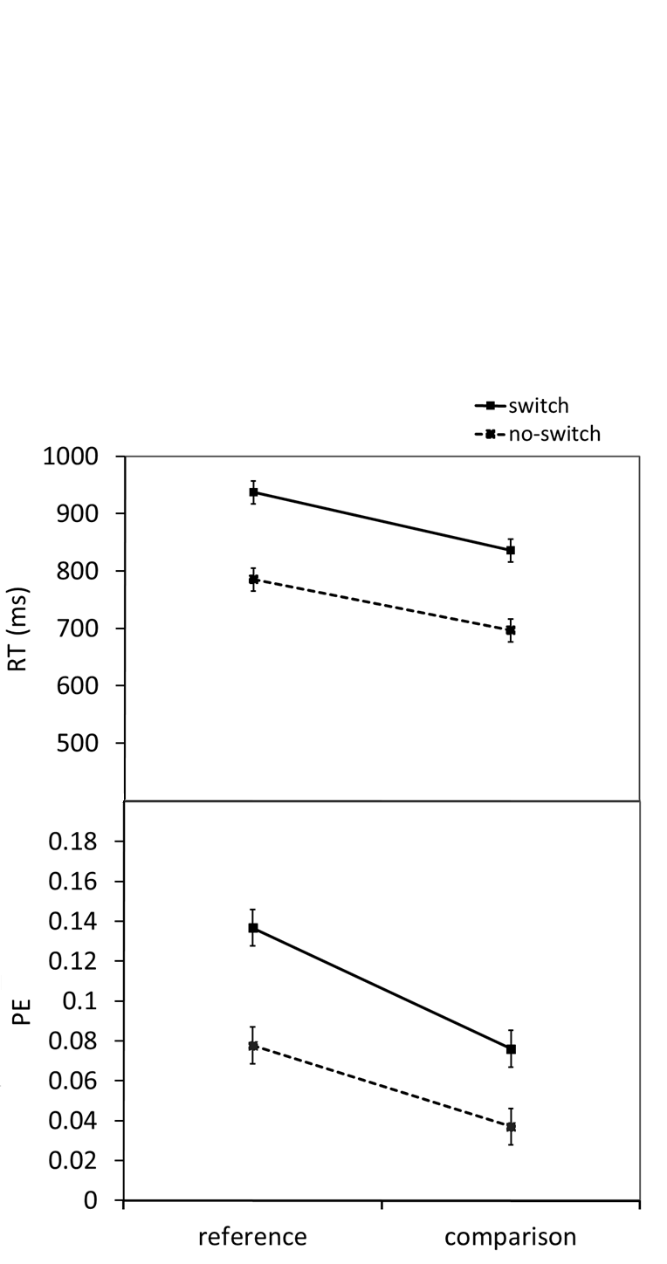


**Figure S4**│ **The behavioral results of Experiment 1**. RT (top), proportion of error (PE) (bottom). Error bars represent 95% confidence intervals.

Experiment 2

To ensure that the increased window size did not alter the behavioral results, a two-way ANOVA was conducted on the RT data with Trial-Type (reference, comparison) and Switching (switch, no-switch) as within-subject independent variables (see Fig. S5). Only the first trial in the sequence of the same trial-type was considered a switch trial. Only correct trials preceded by 2 correct trials were included in this analysis. Both main effects were significant. As before, reference trials were slower than comparison trials, *F(1,19)=24.34, MSe=1,782.89, p<.001, η_p_^2^=.56,* and switch trials were slower than no-switch trials, *F(1,19)=57.41, MSe=2,659.01, p<.001, η_p_^2^=.75*. The two-way interaction was non-significant, *F(1,19)=.02, MSe=2,371, p=.88, η_p_^2^=.00*.

A similar analysis was also done on the PE data. More errors were observed in reference than in comparison trials, *F(1,19)=125.46, MSe=.0008, p<.001, η_p_^2^=.87,* and also more errors were observed on switch than on no-switch trials, *F(1,19)=70.78, MSe=.0010, p<.001, η_p_^2^=.79*. The two-way interaction was significant, *F(1,19)=114.12, MSe=.0008, p<.001, η_p_^2^=.86*, reflecting larger switch cost in reference trials than in comparison trials. Thus, at the behavioral level, the results in Experiment 2 replicated those of Experiment 1.


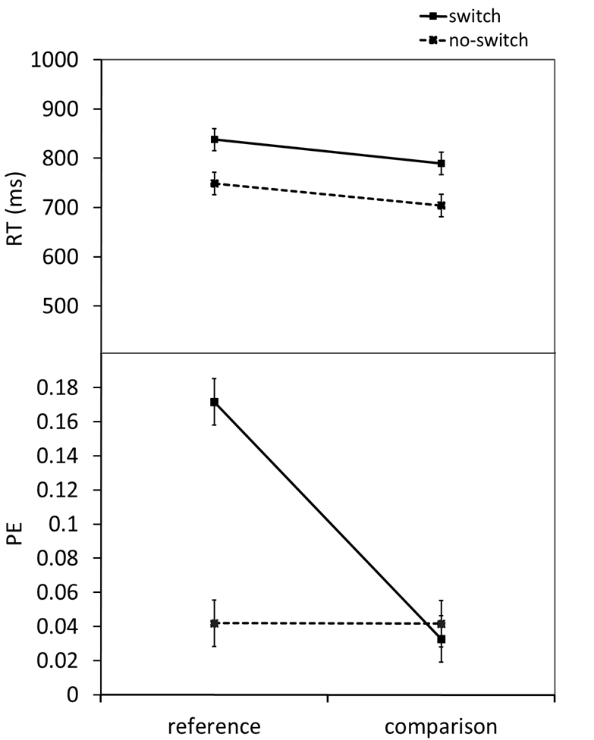


**Figure S5│ The behavioral results of Experiment 2**. RT (top), proportion of error (PE) (bottom). Error bars represent 95% confidence intervals.

Experiment 3

A two-way ANOVA was conducted on the RT data with Trial-Type (reference, comparison) and Switching (switch, no-switch) as within-subject independent variables (Figure 11). Only the first trial in the sequence of the same trial-type was considered a switch trial. Only correct trials preceded by 2 correct trials were included in this analysis. As in Experiments 1 and 2, subjects were slower on switch compared to no-switch trials, as indicated by a significant main effect of Switching, *F(1,20)=14.97, MSe=1,921.07, p<.001, η_p_^2^=.43*. However, this switch cost was smaller (37ms) than the switch cost observed in Experiment 2 (87ms), *F(1,39)=11.39, MSe=2,280.58, p=.002*, *η_p_^2^=.23*.

This difference in switch cost likely reflects the effect of preparation towards switching, induced by the cue in Experiment 3. Participants, were equally fast in reference and comparison trials, as indicated by a non-significant main effect of Trial-Type, *F(1,20)=.22, MSe=2,922.36, p=.64, η_p_^2^=.01.* The two-way interaction was non-significant as well*, F(1,20)=1.76, MSe=2,468.67, p=.20, η_p_^2^=.08*.

The PE analysis revealed both main effects, with more errors in reference than in comparison trials, *F(1,20)=11.18, MSe=.0003, p=.003, η_p_^2^=.36*, but with more errors in no-switch than in switch trials, *F(1,20)=9.03, MSe=.0004, p=.006, η_p_^2^=.31*. The two-way interaction was non-significant, *F(1,20)=2.17, MSe=.0003, p=.16, η_p_^2^=.10*.


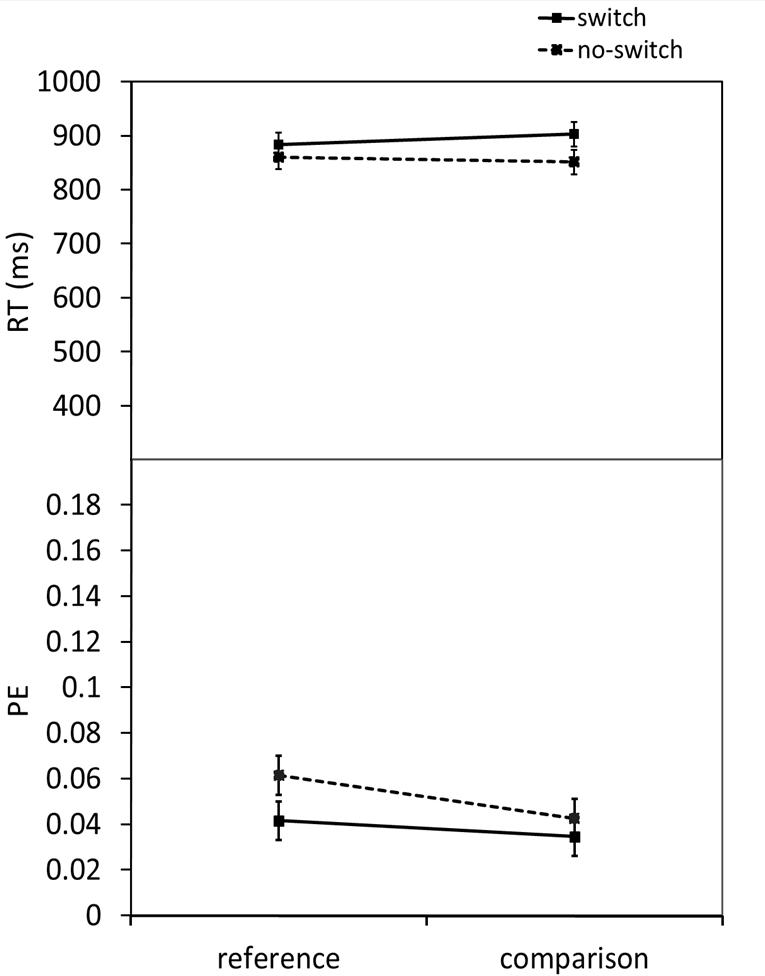
This "reversed" effect for switching between RT and PE (see Fig. S6) could be the results of the long cue presentation time, which might have increased strategy differences between participants in their timing of switching the gate in order to maximize accuracy or to maximize speed of response.

**Figure S6│ The behavioral results of Experiment 3**. RT (top), proportion of error (PE) (bottom). Error bars represent 95% confidence intervals.

**Descriptive statistics of sEBR data**

**Table S3│** Descriptive statistics for the sEBR in resting state before Experiments 1-3.

**Correlation results between sEBR and RT, PE and ebEBR**

**Table S4│** Pearson correlation table between the sEBR in resting state and the effects in the reference-back task as measured with RT, proportion of error (PE) and ebERB. Significant correlations are highlighted in bold.
